# Forkhead box A3 attenuated the progression of fibrosis in a rat model of biliary atresia

**DOI:** 10.1038/cddis.2017.99

**Published:** 2017-03-30

**Authors:** Rui Dong, Yifan Yang, Zhen Shen, Chao Zheng, Zhu Jin, Yanlei Huang, Zhien Zhang, Shan Zheng, Gong Chen

**Affiliations:** 1 Department of Pediatric Surgery, Children's Hospital of Fudan University, and Key Laboratory of Neonatal Disease, Ministry of Health, Shanghai, China

## Abstract

Biliary atresia is a rare, devastating disease of infants where a fibroinflammatory process destroys the bile ducts, leading to fibrosis and biliary cirrhosis, and death if untreated. The cause and pathogenesis remain largely unknown. We tried to investigate factors involved in biliary atresia, especially forkhead box A3 (Foxa3), which might exert a role in the treatment of liver disease. We used RNA sequencing to sequence the whole transcriptomes of livers from six biliary atresia and six choledochal cysts patients. Then, we employed a rat disease model by bile duct ligation (BDL) and adenovirus transduction to address the function of Foxa3 in biliary atresia. We found that tight junction, adherence junction, cell cycle, apoptosis, chemokine singling, VEGF and MAPK signaling pathways were enriched in biliary atresia livers. We showed that Foxa3 expression was notably decreased in liver samples from biliary atresia patients. More importantly, we found that its lower expression predicted a poorer overall survival of biliary atresia patients. Rats that received BDL surgery and Foxa3 expression adenovirus resulted in a significant decrease in the deposition of collagen, and expression of profibrotic cytokines (transforming growth factor-*β* and connective tissue growth factor) and fibrosis markers (*α*-smooth muscle actin, collagen I and collagen III), as compared with rats that received BDL surgery and control adenovirus. Our data suggested a protection role for Foxa3 during the progression of liver fibrosis in biliary atresia, and thereby supported increasing Foxa3 as a targeted treatment strategy.

Biliary atresia is a common cause of neonatal cholestasis characterized by inflammation and bile duct obstruction. It causes cholestasis and progressive liver fibrosis and cirrhosis in infants.^1, 2, 3^ If left untreated, progressive liver cirrhosis leads to liver failure and death by age 2 years. Possible causes of biliary atresia have been proposed, including congenital malformation, congenital cytomegalovirus infection and reovirus 3 infection.^4, 5, 6^ Prognosis of biliary atresia has been significantly improved by Kasai operation, but the majority of patients still need a liver transplant to survive for a long time.^7^

Several studies have performed cDNA microarray to investigate the gene expression profiling of livers from biliary atresia patients.^8, 9, 10^ However, microarrays do have several intrinsic limitations, such as narrow dynamic ranges, low specificity, low sensitivity and hybridization artifacts.^11^ Next-generation RNA sequencing (RNA-Seq) approach exhibits superior sensitivity and capability of detecting splice variants, thus remedying to the above limitations.^12^

In the current study, we used next-generation RNA-Seq to sequence the whole transcriptomes of livers from patients with biliary atresia or control (patients with choledochal cysts (CDCs)). The differential expressed genes were identified between these two groups and the results were then validated by qRT-PCR on several genes. Further, gene set enrichment analysis (GSEA) indicated that the upregulated genes were associated with tight junction, adherence junction, cell cycle, apoptosis, chemokine singling, VEGF and MAPK signaling pathways.

Forkhead box A3 (Foxa3, also known as hepatocyte nuclear factor 3*γ*) is a member of Foxa subfamily of forkhead box proteins, which can bind DNA through a conserved winged-helix- binding motif and act as transcriptional regulators.^13^ Foxa subfamily proteins including Foxa1, Foxa2 and Foxa3 are of high importance in metabolism, organ development and differentiation.^14, 15^ Although either perinatal or embryonic lethality was observed in *Foxa1*- and *Foxa2*-null mice,^16, 17^ *Foxa3*-deficient mice are viable without any obvious abnormalities.^18^ Foxa family proteins are key transcriptional regulators during liver development.^14^ A recent study reported that overexpression of Foxa3 and hepatocyte nuclear factor 4*α* (HNF4*α*) can convert rat bone marrow mesenchymal stem cells to functional hepatocyte-like cells,^19^ which indicates that Foxa3 might exert a role in the treatment of liver disease. Here, among the downregulated genes identified, Foxa3 was confirmed to be downexpressed in biliary atresia livers by western blot and immunohistochemical staining. Further investigation by bile duct ligation (BDL)-induced biliary atresia model indicated that ectopic expression of Foxa3 was significantly attenuated liver fibrosis. Based on our current findings, *Foxa3* could be a promising target gene for biliary atresia therapy owing to its downregulation in biliary atresia livers.

## Results

### Identification of differential expressed genes (DEGs) between biliary atresia and control liver tissues by RNA-seq analysis

**Figure 1:**
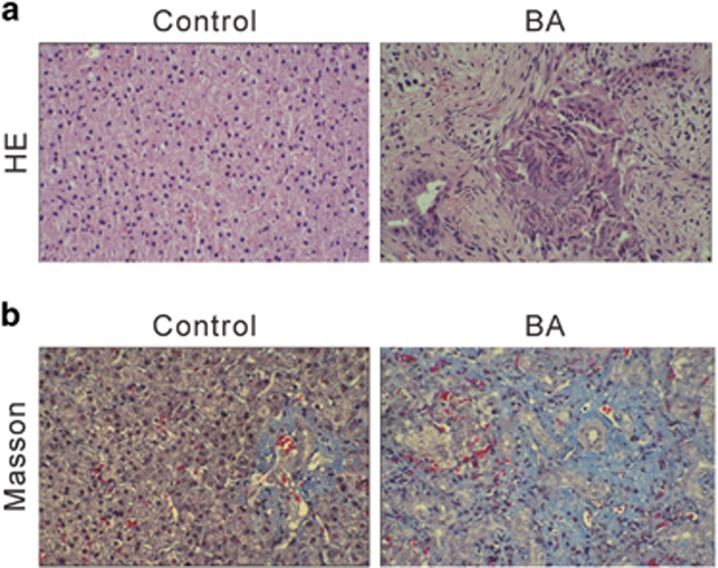
Histological analyses of livers from biliary atresia patients and control. (**a**) HE and (**b**) Masson's trichrome staining

Sixty patients (35 males and 25 females) with type III biliary atresia and 15 CDC patients were enrolled in this study. The CDCs patients with normal liver function were served as control group. Characteristics of the patients are listed in Supplementary Table S1. The serum levels of liver function-related enzymes (alkaline phosphatase, alanine transaminase, aspartate transaminase and gamma-glutamyl transpeptidase), total bile acid, total bilirubin and direct bilirubin were normal in these CDCs patients, but significantly increased in biliary atresia patients. These data revealed the impaired liver function and jaundice in biliary atresia patients. Moreover, representative HE staining and Masson's trichrome showed severe liver fibrosis and collagen accumulation in biliary atresia livers, as compared with control liver (Figure 1).

**Figure 2:**
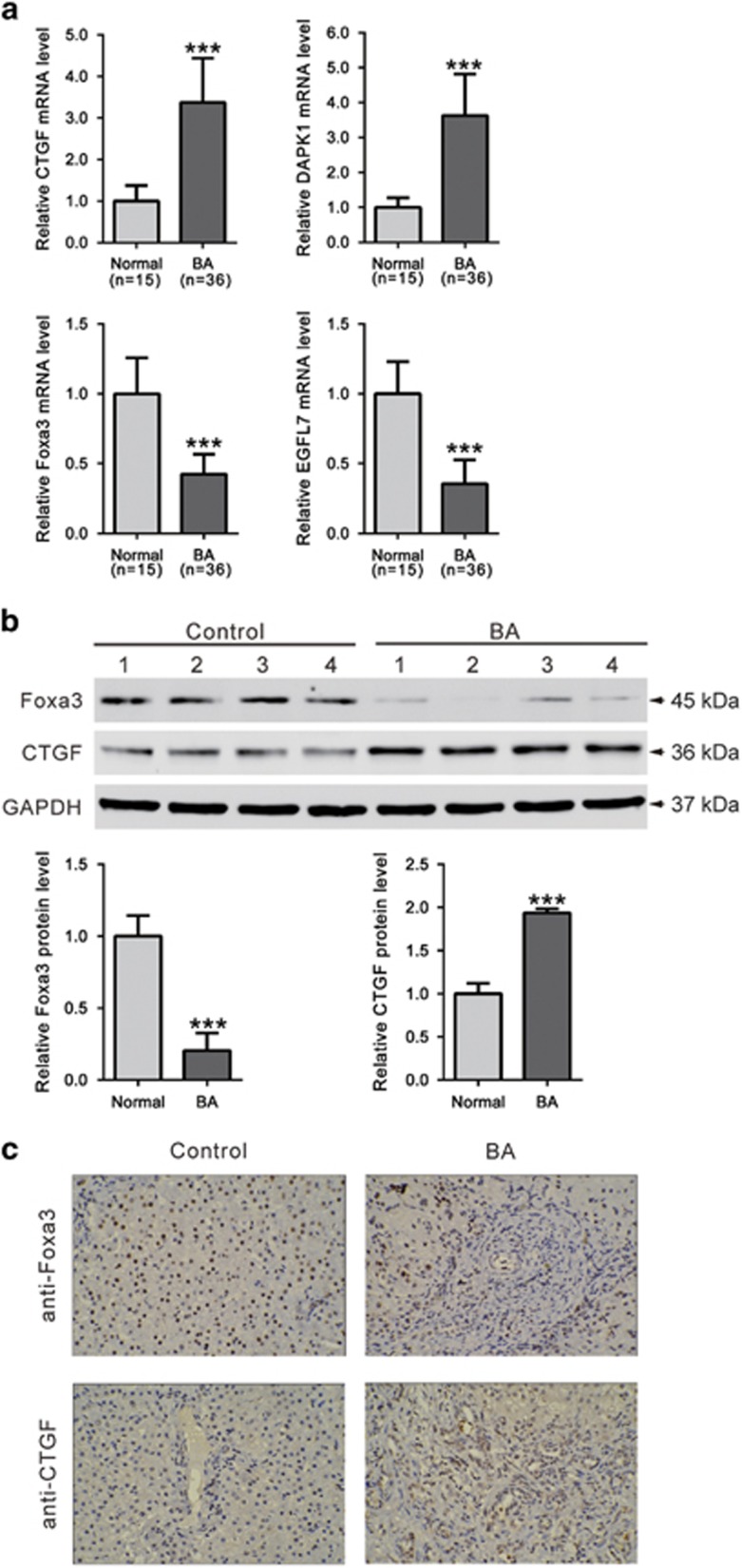
Validation of RNA-seq data. (**a**) mRNA levels of CTGF, DAPK1, Foxa3 and EGFL7 were evaluated by qRT-PCR. Data represent mean values±S.D. from three independent experiments. Protein levels of Foxa3 and CTGF in livers of biliary atresia patients and control were assessed by western blotting (**b**) and immunohistochemistry staining (**c**) (***P<0.001 *versus* control)

We then performed RNA-seq on six pairs of biliary atresia and control liver tissues using the Illumina platform. Genes that exhibited more than 1.5-fold differentially expressed with a *P*-value less than 0.05 were then defined as DEGs. Here, we identified a total of 1751 significantly DEGs with 772 upregulations (Supplementary Table S2) and 979 downregulations (Supplementary Table S3) in biliary atresia liver tissues, when compared with control tissues. The DEGs were functionally related with morphogenesis, fibrogenesis, tissue remodeling, metabolism, cell signal transduction, immunity and so forth. We then carried out GSEA to further determine which biological processes or pathways were involved in biliary atresia. Among 178 KEGG pathways, 31 and 44 pathways were enriched in biliary atresia liver tissues (Table 1) and control tissues (Table 2), respectively (nominal *P*<0.05). It is noteworthy that biliary atresia was positively correlated with multiple genes in tight junction, adherence junction, cell cycle, apoptosis, chemokine singling, VEGF and MAPK signaling pathways, while negatively correlated with multiple genes in bile acid biosynthesis. Validation analysis using qRT-PCR for four DEGs demonstrated the consistent overexpression for connective tissue growth factor (CTGF) and death-associated protein kinase 1 (DAPK1), and the consistent under-expression for Foxa3 and epidermal growth factor like domain 7 (EGFL7) in biliary atresia livers (Figure 2a).

Furthermore, comparing with control livers, a significant increase in protein level of CTGF and a notable decrease in protein level of Foxa3 were observed in biliary atresia livers as measured by western blot (Figure 2b) and immunohistochemistry staining (Figure 2c).

### Downregulation of Foxa3 was associated with the overall survival of biliary atresia

To further explore the functions of Foxa3 on biliary atresia *in vivo*, we established a rat biliary atresia model by BDL. Firstly, we performed RNA-seq on four pairs of BDL and sham-operated liver tissues. A total of 2618 significantly DEGs with 1667 upregulations (Supplementary Table S4) and 951 downregulations (Supplementary Table S5) were identified in BDL liver tissues, when compared with sham-operated liver tissues. We found that 44 DEGs were upregulated, while 62 DEGs were downregulated in both liver samples from biliary atresia patients and BDL rats (Supplementary Figure S1). Four transcription factors, HMGB2, STOH8, CEBPB and Foxa3, were included in the 106 DEGs (Supplementary Table S6). Foxa3 is a member of Foxa family proteins,^20^ which are key transcriptional regulators during liver development.^14^ Overexpression of Foxa3 and HNF4*α* can convert rat bone marrow mesenchymal stem cells to functional hepatocyte-like cells.^19^ Thus, we chose Foxa3 for further investigation.

**Figure 3:**
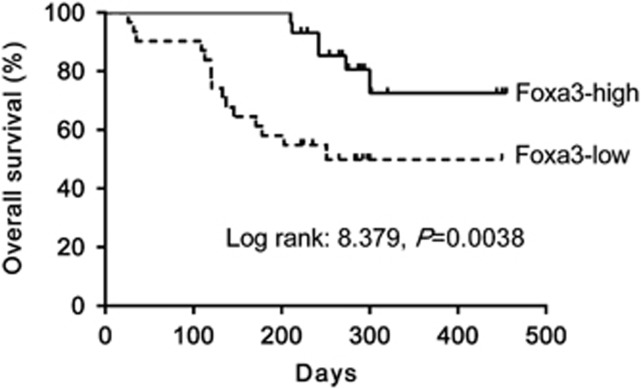
Downregulation of Foxa3 was associated with the overall survival of biliary atresia. Overall survival analysis on 60 biliary atresia patients was performed using the Kaplan–Meier method and log-rank tests

To evaluate the possible prognostic value of Foxa3 on biliary atresia, we detected Foxa3 mRNA expression on all 60 biliary atresia liver samples by qRT-PCR, and divided the patients into Foxa3-high expression group and Foxa3-low expression group by using the median value of Foxa3 mRNA level as a cut-off. We then carried out Kaplan–Meier survival analysis of biliary atresia cases to investigate the clinical outcome of each group. Low expression of Foxa3 was associated with poor overall survival of biliary atresia patients (log-rank=8.379, *P*=0.0038) (Figure 3). By regression analysis, we found that Foxa3 expression had a negative correlation with the degree of fibrosis (*P*<0.001, coefficient of determination *R*=−0.7398), while no obvious correlation was found between age and Foxa3 expression (*P*>0.05).

### Ad5-Foxa3 transduction remarkably attenuated fibrosis in a rat BDL model

**Figure 4:**
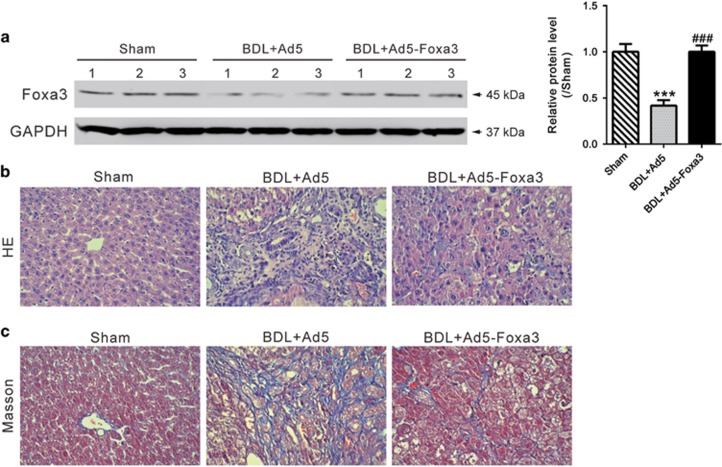
Effect of Ad5-Foxa3 treatment on BDL-induced liver fibrosis. Rats received sham operation or bile duct ligation (BDL) as described in Material and Methods. Two weeks later, rats undergoing BDL surgery were injected with Foxa3-expressing adenovirus (Ad5-Foxa3) or control adenovirus (Ad5). After 2 more weeks, liver tissues were collected. (**a**) Foxa3 expression was evaluated by western blot analysis. Histological analyses were performed by HE (**b**) and Masson's trichrome staining (**c**). BDL surgery caused severe liver fibrosis, while Ad5-Foxa3 injection significantly attenuated BDL-induced liver fibrosis (***P<0.001 *versus* sham-operated group; ^###^*P*<0.001 *versus* BDL+Ad5 group)

**Figure 5:**
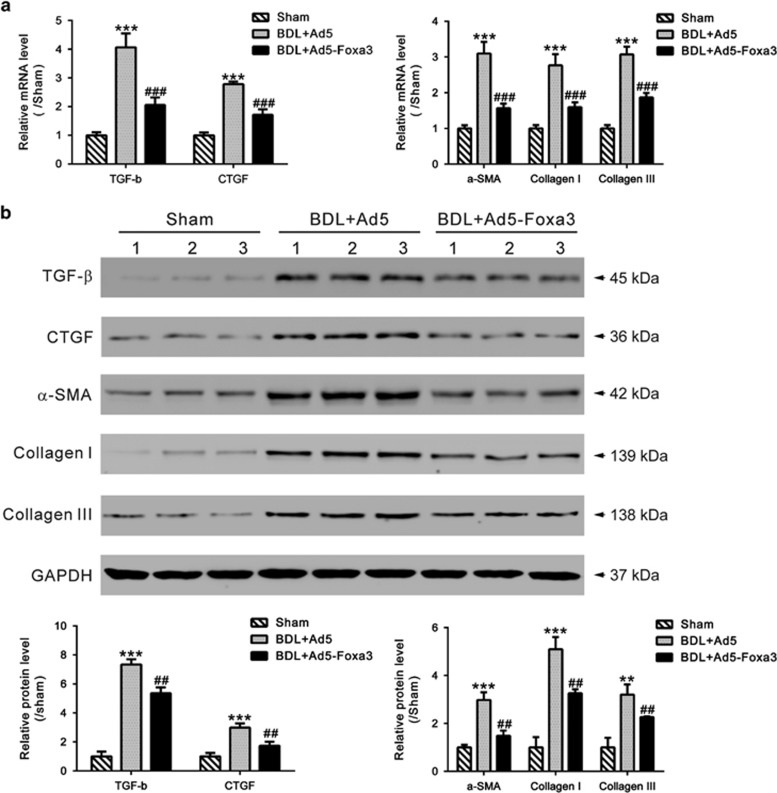
Effect of Ad5-Foxa3 treatment on expression of profibrotic cytokines and fibrosis markers. (**a**) Hepatic mRNA and (**b**) protein levels of TGF-*β*1, CTGF, *α*-SMA, Collagen I and Collagen III were assessed by qRT-PCR and western blotting, respectively (**P<0.01 versus sham-operated group, ***P<0.001 *versus* sham-operated group; ^##^*P*<0.01 versus BDL+Ad5 group,, ^###^*P*<0.001 *versus* BDL+Ad5 group). Data represent mean values±S.D. from three independent experiments

To further emphasize the specific effects of Foxa3, Foxa3-expressing adenoviruses (Ad5-Foxa3) were generated and delivered to the rat BDL model by tail-vein injection 2 weeks after surgery. Four weeks after surgery, liver tissues were collected and western blot was carried out to evaluate Foxa3 expression. Supplementary Figure S2 showed that Foxa3 expression was significantly suppressed by BDL surgery, and rescued by Ad5-Foxa3 injection (Figure 4a). In rats receiving BDL surgery and control adenovirus (Ad5) injection, liver fibrosis and extensive collagen deposition was evident, as shown by HE (Figure 4b) and Masson's trichrome staining (Figure 4c). Injection of Ad5-Foxa3 largely inhibited hepatic collagen accumulation after BDL. These data indicated that Foxa3 can alleviate the fibrosis induced by BDL. The mRNA and protein levels of profibrotic cytokines (TGF-*β*1 and CTGF) and fibrosis markers (*α*-SMA, Collagen I and Collagen III) were then detected (Figure 5). Comparing with sham-operated rats, all detected cytokines and fibrosis markers were significantly increased in livers of BDL rats, which were notably reduced by Ad5-Foxa3 injection. These data suggested the protection effects of Foxa3 on liver fibrosis.

### Expression of Foxa3 and CTGF in rhesus rotavirus (RRV)-induced experimental biliary atresia

**Figure 6:**
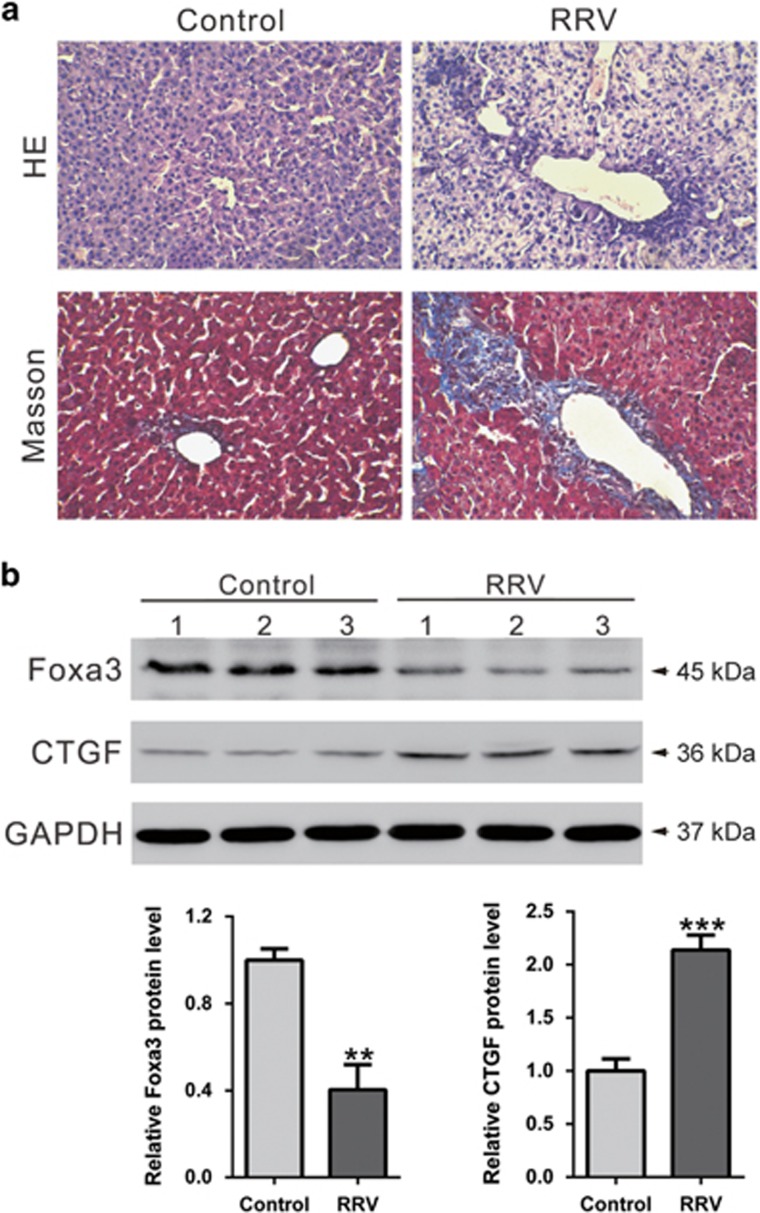
Expression of Foxa3 and CTGF in rhesus rotavirus (RRV)-induced experimental biliary atresia. (**a**) Histological analyses by HE or Masson's trichrome staining. (**b**) Foxa3 and CTGF expression was evaluated by western blot analysis (**P<0.01, ***P<0.001 *versus* Control group)

As a BDL model in rats has little similarity to the biliary atresia, we also established a mouse biliary atresia model by injection with RRV. As shown in Figure 6a, RRV induced severe liver fibrosis and collagen accumulation, as compared with control livers. The changes of Foxa3 and CTGF protein expression were consistent with results observed in human samples.

## Discussion

In this study, we conducted RNA sequencing for six biliary atresia and six CDCs liver tissues. These CDCs patients showed normal liver function as assessed by serum indices and histological analysis, and were served as control in the present study. We analyzed the expression difference at gene levels between BA and control liver tissues and identified 1751 DEGs (Supplementary Tables S2 and S3), many of which were identified as DEGs in previous studies, such as *CFTR* (cystic fibrosis transmembrane conductance regulator), *MMP-7* (matrix metalloproteinase-7), *CTGF*, *LAMC2* (laminin, gamma 2) and *VTCN1* (V-set domain containing T-cell activation inhibitor 1).^8, 21, 22, 23^ We also identified additional novel DEGs, suggesting that the RNA-seq based approach is extremely powerful to study expression profiling.

Biliary atresia is a severe chronic cholestasis disorder of infants. Distinct plasma bile acid profiles were reported in biliary atresia patients.^24^ Recently, increasing evidence has liked the Foxa family of transcription factors, which take part in metabolism, organ development and differentiation,^14^ to bile acid metabolism. Foxa1 and Foxa2 are required for the development of normal bile duct through preventing excess cholangiocyte proliferation.^25^ Bochkis *et al.* reported that hepatocyte-specific knockout of *Foxa2* decreased transcription of genes encoding bile acid transporters, resulting in intrahepatic cholestasis. They also found that Foxa2 was markedly reduced in pediatric subjects with primary sclerosing cholangitis and in those with biliary atresia.^26^ In this study, *Foxa3* was identified as a downregulated gene in biliary atresia livers. Our data showed that Foxa3 was notably decreased in liver samples from biliary atresia patients as evaluated by qRT-PCR, western blotting and immunohistochemistry staining (Figure 2). These findings were further confirmed in BDL (Supplementary Figure S2) and RRV-induced experimental biliary atresia (Figure 6). More importantly, we found that lower expression of Foxa3 predicted a poorer overall survival of biliary atresia patients (Figure 3). We then used the BDL model to explore whether Foxa3 is relevant to the mechanisms of this disease. We found that Foxa3 expressing adenovirus transduction significantly weakened liver fibrosis induced by BDL (Figures 4 and 5). Our data suggested that Foxa3 was a potential prognosis factor for biliary atresia and it may exert antifibrotic effects during the pathogenesis of this disease. However, further investigation is required to figure out how Foxa3 influences profibrotic cytokines and fibrosis markers or whether Foxa3 affects transcription of genes encoding bile acid transporters.

In summary, we reported the expression profile of biliary atresia, and indicated the clinical value of Foxa3 in patients with biliary atresia although there is still long way to go before it can be applied to the clinic.

## Materials and Methods

### Patient samples

This study was approved by the Human Ethics Boards at Children's Hospital of Fudan University. Written informed consent was obtained from the legal guardians of all subjects before starting study procedures. Sixty patients with type III biliary atresia (including 5 patients with advanced cirrosisis) and 15 patients with CDCs who were treated at Children's Hospital of Fudan University from January 2014 to July 2014 were enrolled in this study. Patients with syndromic biliary atresia were excluded. The clinical characteristics of the patients are presented in Supplementary Table S1. All biliary atresia patients underwent successful Kasai portoenterostomy and follow-up lasted for one year. The age at diagnosis was 2.1±0.6 months. None of the patients received liver transplant within one year after surgery. All the samples collected at the time of Kasai portoenterostomy. For RNA and protein extraction, tissue samples were immediately snap-frozen and stored at −80 °C. Liver biopsies were scored for fibrosis stage according to the Metavir score system, which classifies fibrosis according to a 5-point scale: F0, no fibrosis; F1, portal fibrosis without septa; F2, portal fibrosis with few septa; F3, numerous septa without cirrhosis; F4, cirrhosis. Regression analysis was carried out to determine the relationship between the degree of fibrosis and Foxa3 by using Medcalc software (MedCalc, Ostend, Belgium).

### RNA extraction, processing and sequencing

Total RNA was extracted from liver tissues using the Trizol reagent (Invitrogen, Carlsbad, CA, USA) according to the manufacturer's protocol. Extracted RNA was quantified by using an ND-1000 Spectrophotometer (NanoDrop Technologies, Wilmington, DE, USA). RNA integrity was assessed by denaturing formaldehyde gel electrophoresis. RNA-Seq libraries were prepared by using Illumina's TruSeq Sample Preparation Kit. Briefly, polyA-containing messenger RNA was captured from 10 *μ*g of total RNA, fragmented into small fragments, and reverse-transcribed into cDNA. The cDNA was fragmented and ligated to adapters. The cDNA libraries was then created by using 15 cycles of PCR. Each sample was cleaned up on an RNeasy Mini Column (Qiagen, Limburg, Netherlands), treated with DNase and analyzed for quality on an Agilent 2100 Bioanalyzer (Agilent Technologies, Santa Clara, CA, USA). Samples were on an Illumina HiSeq 2000 for 2 × 100-bp paired-end sequencing. Reads were mapped to the human genome (hg19) using TopHat v2.0.11 (http://tophat.cbcb.umd.edu)^27^ with the following default options with a TopHat transcript index built from Ensembl_GRCh37. Transcript expression was estimated with an improved version of Cuffdiff2 (http://cufflinks.cbcb.umd.edu).^28^ Cuffdiff was run with the default options against the UCSC iGenomes GTF file from Illumina (available at http://cufflinks.cbcb.umd.edu/igenomes.html). The workflow used to analyze the data is described in detail in Trapnell *et al*.^29^ To identify a gene or transcript as differential expression, Cuffdiff2 tests the observed log-fold-change in its expression against the null hypothesis of no change (i.e., the true log-fold-change is zero). Clustering of gene expression profiles was achieved with the csDendro function from CummeRbund (http://compbio.mit.edu/cummeRbund/).

All of our original sequence data have been deposited in NCBI's Sequence Read Archive database (http://www.ncbi.nlm.nih.gov/sra, AC: SRA297629 and SRP063995).

### Pathway analysis and bioinformatics

To identify the pathways that were significantly enriched in samples from biliary atresia patients or controls, GSEA was performed as describe previously^30^ using a total of 178 gene sets from Kyoto Encyclopedia of Genes and Genomes (KEGG).The gene sets showing FDR, 0.25, a well-established cutoff for the identification of biologically relevant genes, were considered enriched between the classes under comparison.

### Histology and immunohistochemistry

Liver specimens were fixed in 10% neutral-buffered formalin and embedded in paraffin. The embedded tissues were cut into 5 *μ*m-thick serial sections. The sections were deparaffinized in xylene, hydrated through graded ethanol and stained with hematoxylin & eosin (HE) or Masson's trichrome. For immunohistochemistry staining, deparaffinized and hydrated sections were rinsed in PBS and treated with 0.3% hydrogen peroxide for blocking endogenous peroxidase activity. After probed with primary antibodies (antibodies against CTGF and Foxa3 were from Abcam (Cambridge, UK) and Santa Cruz (Santa Cruz, CA, USA), respectively) at 4 °C overnight, the sections were incubated with appropriate horseradish peroxidase-conjugated secondary antibodies (Longisland Biotech., Shanghai, China) at room temperature for 1 h, and visualized with DAB substrate (Longisland Biotech.) followed by counterstain with hematoxylin. The optimal antibody concentration was determined for each assay with a titration experiment. Negative controls were performed with primary antibody absent and mouse or rabbit isotype control antibody.

### Quantitative RT-PCR analysis

Total RNA was isolated from snap-frozen liver tissues using TRIzol Reagent (Invitrogen) followed by DNase treatment (Promega, Madison, WI, USA). Complementary DNA was prepared by reverse transcription with M-MLV reverse transcriptase (Fermentas, Hanover, MD, USA) according to the manufacturers' instructions. Quantitative RT-PCR was performed on an ABI 7300 Sequence Detection System (Applied Biosystems, Foster City, CA, USA) with SYBR Green PCR mix (Thermo Fisher Scientific, Rockford, IL, USA). Primers used are summarized in Supplementary Table S7. Gene expression values were calculated using the ΔΔ Ct method^31^ and GAPDH was served as an endogenous control.

### Western blotting

Snap-frozen liver samples were homogenized in radioimmunoprecipitation assay buffer. Protein concentration was determined by BCA protein assay (Thermo Fisher Scientific). Equal amounts of protein were subjected to SDS-PAGE gels following with electrophoretic transfer to nitrocellulose membranes. The membranes were blocked with 5% nonfat milk, and incubated with the primary antibody for Foxa3 (sc-25357; Santa Cruz), transforming growth factor-*β* (TGF-*β*, Ab64715; Abcam), CTGF (Ab6992; Abcam), Collagen I (Ab34710; Abcam), Collagen III (Ab7778; Abcam), *α*-smooth muscle actin (*α*-SMA, #14968; Cell Signaling Technology, Danvers, MA, USA) or GAPDH (#5174, Cell Signaling Technology). After incubated with corresponding horseradish peroxidase-coupled secondary antibody (Beyotime, Shanghai, China), the membrane was developed with enhanced chemiluminescence system (Bio-Rad, Richmond, CA, USA). Densitometric analysis was performed by using ImageJ software (National Institutes of Health, Bethesda, MD, USA) using GAPDH as an endogenous control.

### Construction of the recombinant adenovirus Ad5-Foxa3

Foxa3 expression plasmid (PDC315-Foxa3) and a LacZ-containing control plasmid (PDC315-LacZ) were constructed and confirmed by sequencing. PDC315-Foxa3 or PDC315-LacZ was then transfected into HEK293 cells with pBGHE3 (Microbix Biosystems, Mississauga, Ontario, Canada) by using Lipofectamine 2000 (Invitrogen) according to the protocol provided by the manufacturer. Recombinant adenovirus Ad5-Foxa3 or Ad5-LacZ was grown in HEK293 cells and purified on a cesium chloride gradient. After viral titers were determined by plaque assay, virus was aliquoted and stored at −80 °C.

Rat disease model induced by BDL.The experiments were carried out according to the guidelines of the Ethics Committee of Fudan University. Six-week-old male Sprague-Dawley rats, weighing between 180 and 200 g, were purchased from Shanghai Lab Animal Research Center (Shanghai, China) and kept on the same diet. Eighteen rats were randomly divided into three groups: group I, sham operated (control); group II, BDL+Ad5-LacZ; group III, BDL+Ad5-Foxa3. All surgical procedure was performed under strict sterile conditions as previously described.^32^ All rats were anesthetized by intraperitoneal injection with pentobarbital sodium (50 mg/kg). Laparotomy was performed on all rats via an approximately 1 cm upper-midline incision to identify the bile duct. In group II and group III, the common bile duct was isolated, doubly ligated and transected between the ligatures. In group I, no ligation or resection was performed. Two weeks after BDL, recombinant adenovirus Ad5-LacZ and Ad5-Foxa3 (5 × 10^9^ PFU per rat, 0.5 ml) were tail-vein injected into rats of group II and group III, respectively. Four weeks after surgery, rats were killed at 0900–0930 hours and killed, and liver tissues were then collected. For histological analysis, liver samples were fixed in 10% buffered formalin, embedded in paraffin and cut into 5-*μ*m slices; for RNA or protein extraction, liver samples were snap-frozen and stored at −80 °C.

### Mouse biliary atresia model induced by RRV

Healthy Balb/c pregnant maternal mice were purchased from SLAC Inc. (Shanghai, China) and isolated in laminar-flow cages. Newborn Balb/c mice were randomized into two groups in proportion of 2 : 1 (experimental group:control group). Pups in the experimental group were inoculated intraperitoneally with 25 *μ*l minimum essential medium, containing 10^6^ PFU of RRV. Control group received 25 *μ*l 2% FCS-MEM. Pups that died due to infection, did not feed or that were cannibalized by their mothers were excluded from further analysis. All mice were weighed every 2 days and observed for signs of cholestasis (icterus of the non-fur-covered skin, color and quality of stools, and the appearance of bilirubin in the urine) until 14 days when they were killed. Liver samples were collected for histological analysis and western blotting analysis.

### Statistical analysis

Statistical analyses were performed in GraphPad Prism v6.0 software (GraphPad Software Inc., La Jolla, CA, USA). Data were expressed as mean±S.D. For survival analysis, overall survival was defined as the time interval between the date of operation to the date of death or the last follow-up. The prognostic significance analysis was performed using the Kaplan–Meier method and log-rank tests. Statistical significance between two groups was determined by Student's *t-*test. Statistically significant differences were defined as having a *P*-value less than 0.05.

## Figures and Tables

**Table 1:** Statistically significant KEGG classifications of enrichment in biliary atresia patients

**KEGG subcategories**	**Size**	***P*****-value**	**ES**
FC_GAMMA_R_MEDIATED_PHAGOCYTOSIS	96	0.0000	0.4904
CELL_CYCLE	124	0.0000	0.4553
PANCREATIC_CANCER	70	0.0000	0.4754
UBIQUITIN_MEDIATED_PROTEOLYSIS	132	0.0000	0.4414
PATHWAYS_IN_CANCER	320	0.0010	0.3949
T_CELL_RECEPTOR_SIGNALING_PATHWAY	107	0.0010	0.4608
SMALL_CELL_LUNG_CANCER	84	0.0020	0.4556
ENDOMETRIAL_CANCER	52	0.0021	0.5053
COLORECTAL_CANCER	62	0.0031	0.4647
HEDGEHOG_SIGNALING_PATHWAY	53	0.0064	0.4819
AXON_GUIDANCE	128	0.0080	0.3976
ADHERENS_JUNCTION	73	0.0083	0.4452
NON_SMALL_CELL_LUNG_CANCER	54	0.0096	0.4716
TIGHT_JUNCTION	128	0.0101	0.4003
ACUTE_MYELOID_LEUKEMIA	57	0.0105	0.4580
SPLICEOSOME	125	0.0121	0.3977
CHRONIC_MYELOID_LEUKEMIA	73	0.0145	0.4225
PHOSPHATIDYLINOSITOL_SIGNALING_SYSTEM	75	0.0185	0.4160
NEUROTROPHIN_SIGNALING_PATHWAY	124	0.0212	0.3798
CHEMOKINE_SIGNALING_PATHWAY	180	0.0261	0.3565
HOMOLOGOUS_RECOMBINATION	28	0.0284	0.5023
BASAL_CELL_CARCINOMA	53	0.0335	0.4334
ERBB_SIGNALING_PATHWAY	87	0.0372	0.3939
APOPTOSIS	84	0.0379	0.3974
THYROID_CANCER	29	0.0394	0.4947
NATURAL_KILLER_CELL_MEDIATED_CYTOTOXICITY	118	0.0394	0.3762
VEGF_SIGNALING_PATHWAY	72	0.0404	0.4117
MAPK_SIGNALING_PATHWAY	256	0.0410	0.3409
BASAL_TRANSCRIPTION_FACTORS	33	0.0416	0.4796
LEISHMANIA_INFECTION	70	0.0487	0.4022
LEUKOCYTE_TRANSENDOTHELIAL_MIGRATION	110	0.0491	0.3799

**Table 2:** Statistically significant KEGG classifications of enrichment in normal control

**KEGG subcategories**	**Size**	***P*****-value**	**ES**
RETINOL_METABOLISM	61	0.0000	−0.6814
METABOLISM_OF_XENOBIOTICS_BY_CYTOCHROME_P450	65	0.0000	−0.6900
FATTY_ACID_METABOLISM	42	0.0000	−0.7198
DRUG_METABOLISM_CYTOCHROME_P450	67	0.0000	−0.6611
VALINE_LEUCINE_AND_ISOLEUCINE_DEGRADATION	44	0.0000	−0.6638
RIBOSOME	85	0.0000	−0.5872
COMPLEMENT_AND_COAGULATION_CASCADES	67	0.0000	−0.5820
PRIMARY_BILE_ACID_BIOSYNTHESIS	16	0.0000	−0.8517
STEROID_HORMONE_BIOSYNTHESIS	52	0.0000	−0.6167
OXIDATIVE_PHOSPHORYLATION	112	0.0000	−0.5179
PROPANOATE_METABOLISM	32	0.0000	−0.6790
TRYPTOPHAN_METABOLISM	39	0.0000	−0.6453
PARKINSONS_DISEASE	110	0.0000	−0.5182
PEROXISOME	78	0.0000	−0.5082
LINOLEIC_ACID_METABOLISM	26	0.0000	−0.6252
GLYCINE_SERINE_AND_THREONINE_METABOLISM	31	0.0000	−0.6368
TYROSINE_METABOLISM	39	0.0000	−0.5651
GLYCOLYSIS_GLUCONEOGENESIS	59	0.0000	−0.4957
GLYOXYLATE_AND_DICARBOXYLATE_METABOLISM	15	0.0000	−0.7207
PENTOSE_AND_GLUCURONATE_INTERCONVERSIONS	26	0.0000	−0.6133
PROTEASOME	44	0.0000	−0.5106
DRUG_METABOLISM_OTHER_ENZYMES	49	0.0000	−0.4956
PORPHYRIN_AND_CHLOROPHYLL_METABOLISM	38	0.0000	−0.5217
ASCORBATE_AND_ALDARATE_METABOLISM	23	0.0000	−0.5724
BETA_ALANINE_METABOLISM	21	0.0000	−0.5847
PPAR_SIGNALING_PATHWAY	65	0.0000	−0.4127
ARACHIDONIC_ACID_METABOLISM	55	0.0000	−0.4299
PYRUVATE_METABOLISM	39	0.0000	−0.4370
TERPENOID_BACKBONE_BIOSYNTHESIS	15	0.0000	−0.5692
RENIN_ANGIOTENSIN_SYSTEM	16	0.0000	−0.5532
ONE_CARBON_POOL_BY_FOLATE	17	0.0000	−0.5381
BUTANOATE_METABOLISM	32	0.0000	−0.4446
HUNTINGTONS_DISEASE	167	0.0000	−0.4495
CITRATE_CYCLE_TCA_CYCLE	29	0.0000	−0.3206
ARGININE_AND_PROLINE_METABOLISM	54	0.0000	−0.4296
ALZHEIMERS_DISEASE	154	0.0000	−0.3626
GLUTATHIONE_METABOLISM	48	0.0000	−0.3144
PRION_DISEASES	34	0.0000	−0.3474
NEUROACTIVE_LIGAND_RECEPTOR_INTERACTION	222	0.0000	−0.3628
PENTOSE_PHOSPHATE_PATHWAY	26	0.0078	−0.3808
HISTIDINE_METABOLISM	28	0.0263	−0.3546
NICOTINATE_AND_NICOTINAMIDE_METABOLISM	24	0.0348	−0.3149
STARCH_AND_SUCROSE_METABOLISM	46	0.0357	−0.3833
PYRIMIDINE_METABOLISM	98	0.0417	−0.2991
