# Supplementary material for: Forkhead box A3 attenuated the progression of fibrosis in a rat model of biliary atresia
Source: Cell Death Dis. 2017 Mar 30;8(3):e2719–. doi: 10.1038/cddis.2017.99 (PMC5386589; doi:10.1038/cddis.2017.99)
Supplement: Supplementary Information [file cddis201799x1.docx]

**Supplementary Figure Legends**

Supplementary Figure 1. Venn diagram.

Supplementary Figure 2. Foxa3 expression in BDL-induced liver fibrosis. (*A*) Foxa3 expression was evaluated by western blot analysis. Histological analyses by (*B*) HE and (*C*)Masson’s trichrome staining.
