# Supplementary figures and images for: Forkhead box A3 attenuated the progression of fibrosis in a rat model of biliary atresia
Source: Cell Death Dis. 2017 Mar 30;8(3):e2719–. doi: 10.1038/cddis.2017.99 (PMC5386589; doi:10.1038/cddis.2017.99)

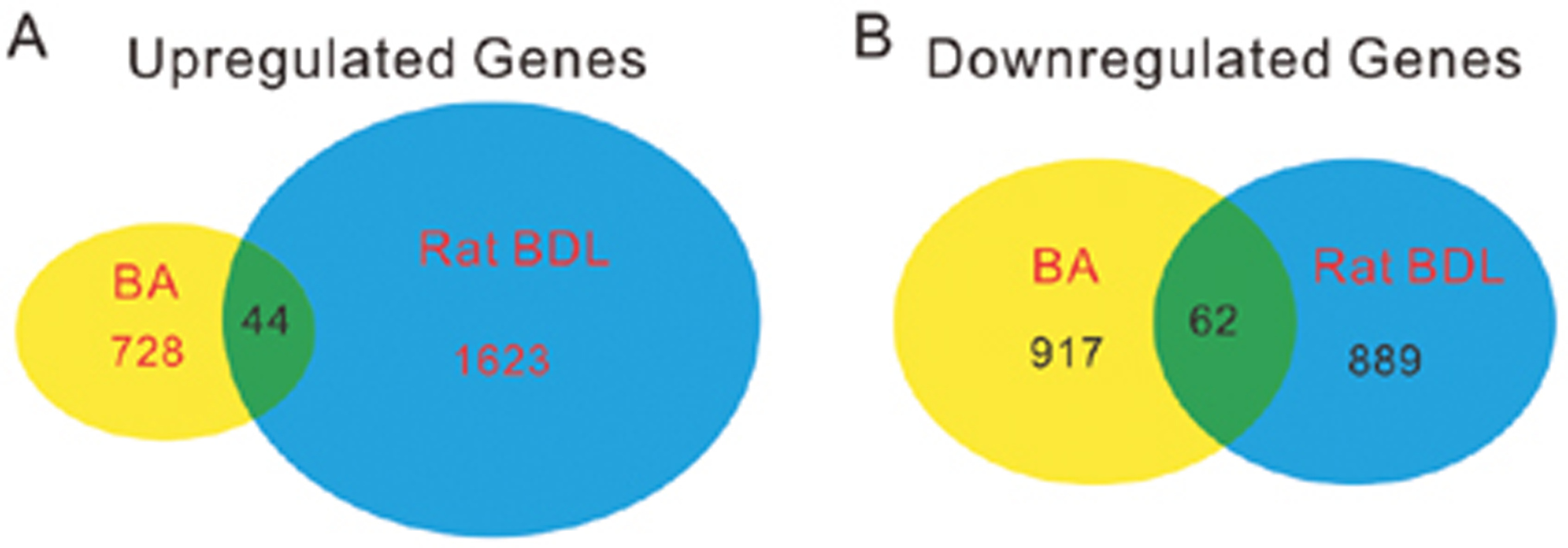

Supplement: Supplementary Figure 1 [file cddis201799x2.tif]

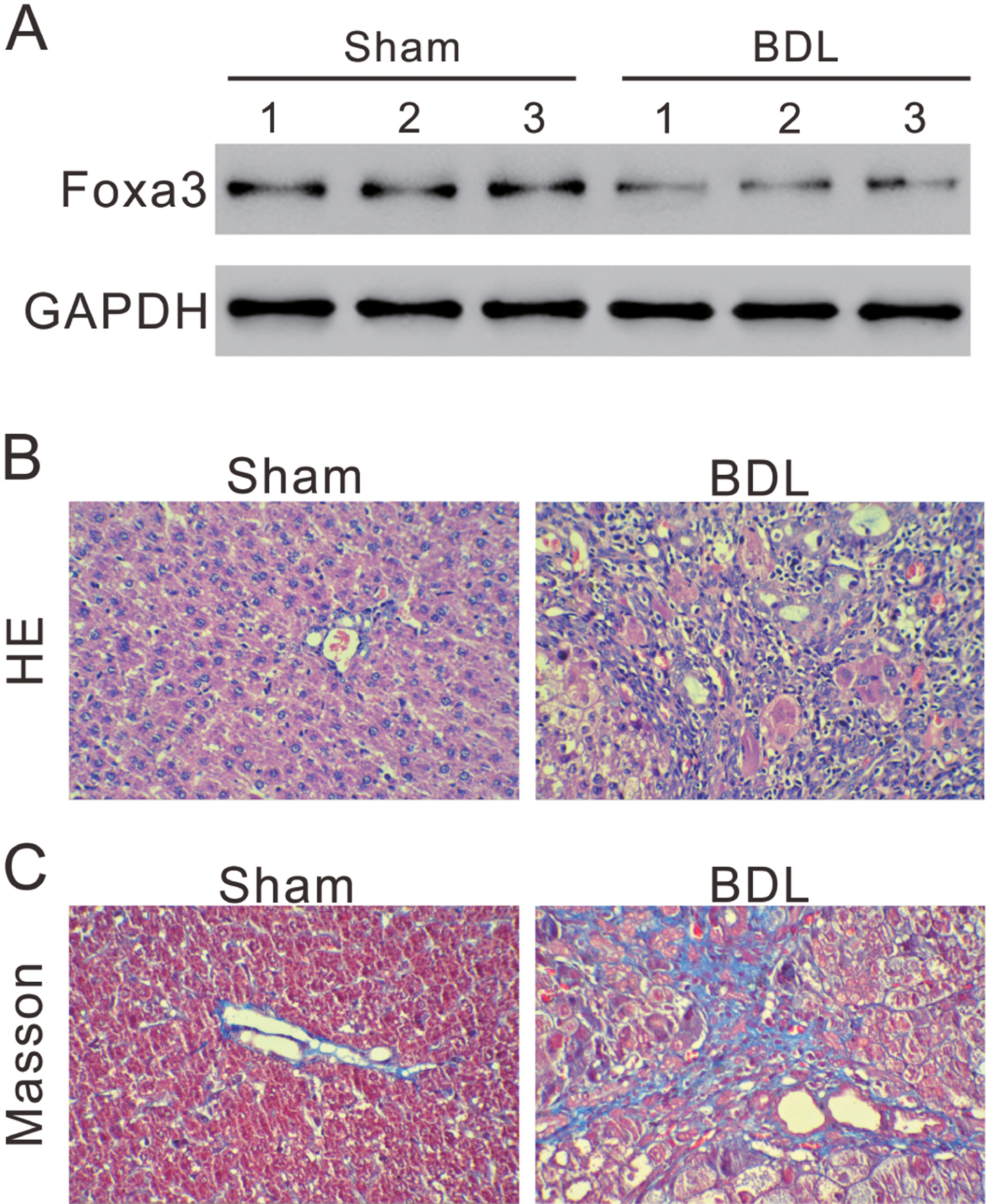

Supplement: Supplementary Figure 2 [file cddis201799x3.tif]
