# Supplementary material for: Forkhead box A3 attenuated the progression of fibrosis in a rat model of biliary atresia
Source: Cell Death Dis. 2017 Mar 30;8(3):e2719–. doi: 10.1038/cddis.2017.99 (PMC5386589; doi:10.1038/cddis.2017.99)
Supplement: Supplementary Table 1 [file cddis201799x4.docx]

**Supplementary Table 1.** Characteristics of patients with biliary atresia or choledochal cysts.

|  | BA | CDCs | *P*-value |
| --- | --- | --- | --- |
| Age^a^ (months) | 2.1 ± 0.6 | 18.7 ± 3.5 | >0.05 |
| Male | 35 | 8 | N/A |
| Female | 25 | 7 | N/A |
| Diagnosis Type | III^b^ | I^c^ | N/A |
| ALP (IU/L) | 625.5 ± 28.7 | 77.6 ± 12.4 | <0.05 |
| ALT (IU/L) | 122.6 ± 20.2 | 12.5 ± 5.6 | <0.05 |
| AST (IU/L) | 176.4 ± 22.5 | 18.3 ± 7.2 | <0.05 |
| DBIL (umol/L) | 121.1 ± 6.2 | 3.2 ± 1.2 | <0.05 |
| TBIL (umol/L) | 145.1 ± 12.2 | 7.5 ± 2.3 | <0.05 |
| GGT (IU/L) | 686.8 ± 86.4 | 36.4 ± 13.6 | <0.05 |
| TBA (umol/L) | 126.2 ± 8.2 | 7.6 ± 2.2 | <0.05 |

^a^ At operation day.

^b^ Type III atresia refers to the discontinuity of both right and left hepatic ducts to the level of the porta hepatis.

^c^ Type I is the most common (80-90%) involving saccular or fusiform dilatation of a portion or entire common bile duct with normal intrahepatic duct. CDCs patients without jaudice have normal liver function as controls.
