# Supplementary material for: Forkhead box A3 attenuated the progression of fibrosis in a rat model of biliary atresia
Source: Cell Death Dis. 2017 Mar 30;8(3):e2719–. doi: 10.1038/cddis.2017.99 (PMC5386589; doi:10.1038/cddis.2017.99)
Supplement: Supplementary Table 6 [file cddis201799x9.docx]

**Supplementary Table 6.** DEGs identified in BA patients and BDL rats.

| Gene | Human | | Mouse | |
| --- | --- | --- | --- | --- |
|  | log2(Control/BA) | p_value | log2(Sham/BDL) | p_value |
| Up-regulated genes |  |  |  |  |
| ABCG5 | -0.881704 | 0.04005 | -0.856399 | 5.00E-05 |
| ABHD3 | -1.52673 | 0.0331 | -5.37973 | 0.0161 |
| ACOT12 | -1.03124 | 0.0089 | -0.882575 | 5.00E-05 |
| ANKRD49 | -1.15974 | 0.02295 | -0.695449 | 5.00E-05 |
| ANXA1 | -1.32936 | 0.02165 | -0.980352 | 5.00E-05 |
| ANXA3 | -2.01812 | 0.00075 | -0.96621 | 5.00E-05 |
| CCNC | -0.909869 | 0.0174 | -1.05498 | 5.00E-05 |
| CCNL2 | -0.980865 | 0.02025 | -1.27212 | 5.00E-05 |
| CD53 | -1.47606 | 0.00395 | -1.47889 | 5.00E-05 |
| CYTIP | -1.42425 | 0.0003 | -0.965194 | 0.0001 |
| DDX59 | -1.31349 | 0.033 | -0.62642 | 0.00195 |
| DHX40 | -0.775194 | 0.034 | -1.08058 | 5.00E-05 |
| DNAJC10 | -1.03215 | 0.01565 | -0.780962 | 5.00E-05 |
| ELOVL2 | -1.60278 | 0.0006 | -0.809243 | 5.00E-05 |
| EPB41L1 | -0.718557 | 0.04485 | -0.696526 | 5.00E-05 |
| EPCAM | -2.7244 | 5.00E-05 | -0.738077 | 5.00E-05 |
| ERO1L | -1.30915 | 0.0023 | -0.815451 | 5.00E-05 |
| FAM111A | -0.740382 | 0.04645 | -8.90019 | 0.00015 |
| FMO1 | -1.55693 | 0.0392 | -0.985203 | 5.00E-05 |
| HMGB2 | -1.17642 | 0.02755 | -0.924589 | 5.00E-05 |
| HNRNPK | -1.31535 | 0.0008 | -0.602481 | 5.00E-05 |
| HOPX | -1.66752 | 0.01915 | -2.47146 | 0.0004 |
| KIF12 | -0.902616 | 0.0402 | -0.634055 | 0.0032 |
| LGALS3 | -1.08021 | 0.02375 | -0.697645 | 5.00E-05 |
| LPXN | -1.17186 | 0.0121 | -0.815054 | 0.0014 |
| MCAM | -0.909814 | 0.02315 | -0.599808 | 0.00155 |
| MOSPD1 | -1.14008 | 0.01605 | -0.903043 | 5.00E-05 |
| MYL12A | -0.991592 | 0.02565 | -1.23466 | 5.00E-05 |
| NIPAL2 | -1.24591 | 0.0018 | -1.17807 | 5.00E-05 |
| PPIL3 | -1.05106 | 0.0159 | -1.208 | 5.00E-05 |
| PTPRC | -1.79986 | 0.0001 | -0.82719 | 5.00E-05 |
| RANBP17 | -1.68599 | 0.00515 | -2.87758 | 5.00E-05 |
| RGS1 | -1.54197 | 0.00265 | -0.851224 | 0.0004 |
| S100A11 | -0.798046 | 0.04475 | -1.31946 | 5.00E-05 |
| SERPINE2 | -1.19849 | 0.0173 | -1.00029 | 5.00E-05 |
| SLC25A33 | -0.972158 | 0.014 | -1.31939 | 5.00E-05 |
| SMC4 | -2.04647 | 0.00085 | -0.833952 | 5.00E-05 |
| SPIN1 | -1.36148 | 0.0039 | -0.927518 | 5.00E-05 |
| STMN1 | -1.37761 | 0.00155 | -1.2361 | 5.00E-05 |
| TLE1 | -1.03961 | 0.02495 | -0.716007 | 5.00E-05 |
| VCAM1 | -0.898635 | 0.04455 | -1.23987 | 5.00E-05 |
| WEE1 | -1.54501 | 0.00235 | -3.44616 | 5.00E-05 |
| XPO1 | -1.04481 | 0.0027 | -0.684253 | 5.00E-05 |
| YWHAZ | -1.0304 | 0.0211 | -0.74858 | 5.00E-05 |
| Downregulated genes |  |  |  |  |
| ACACB | 1.71895 | 0.0005 | 2.00278 | 5.00E-05 |
| ACSM5 | 1.23487 | 0.00205 | 3.62853 | 5.00E-05 |
| ACY3 | 1.09274 | 0.02015 | 0.68789 | 5.00E-05 |
| ADCK3 | 0.877919 | 0.03055 | 1.49023 | 5.00E-05 |
| AGXT2 | 2.05787 | 5.00E-05 | 1.30131 | 5.00E-05 |
| AOX1 | 3.59799 | 5.00E-05 | 1.50251 | 5.00E-05 |
| APOF | 4.32871 | 5.00E-05 | 0.732122 | 5.00E-05 |
| ATOH8 | 1.21056 | 0.01935 | 1.57309 | 5.00E-05 |
| BCKDK | 1.17201 | 0.00365 | 0.736048 | 5.00E-05 |
| BCL3 | 1.03379 | 0.0041 | 0.957237 | 5.00E-05 |
| BLVRB | 1.45309 | 0.0013 | 0.80117 | 5.00E-05 |
| C6 | 1.13739 | 0.0082 | 0.877808 | 5.00E-05 |
| CD82 | 1.84045 | 5.00E-05 | 1.06542 | 5.00E-05 |
| CEBPB | 0.852406 | 0.0427 | 0.97253 | 5.00E-05 |
| COPZ2 | 1.55119 | 0.01255 | 0.623262 | 5.00E-05 |
| CYP8B1 | 1.10401 | 0.01865 | 0.733789 | 5.00E-05 |
| DES | 2.06103 | 0.00175 | 0.622066 | 0.0015 |
| EBP | 1.18178 | 0.0058 | 1.10446 | 5.00E-05 |
| EGFL7 | 1.22307 | 0.00955 | 1.41307 | 5.00E-05 |
| FDFT1 | 1.07635 | 0.0052 | 0.881177 | 5.00E-05 |
| FOXA3 | 1.26405 | 0.0024 | 0.560311 | 5.00E-05 |
| FUOM | 1.56175 | 0.0046 | 1.30934 | 5.00E-05 |
| GCAT | 1.91791 | 0.0005 | 1.16831 | 5.00E-05 |
| GCK | 5.5044 | 0.03095 | 1.25959 | 5.00E-05 |
| HFE2 | 0.849918 | 0.0359 | 1.27895 | 5.00E-05 |
| HSD3B7 | 1.2676 | 0.00265 | 0.860514 | 5.00E-05 |
| KCNK5 | 2.77908 | 0.0013 | 0.993573 | 5.00E-05 |
| LAMTOR2 | 1.46942 | 0.00105 | 0.590503 | 5.00E-05 |
| LIMS2 | 1.65799 | 0.0021 | 1.94294 | 5.00E-05 |
| MID1IP1 | 1.38621 | 0.0024 | 1.12024 | 5.00E-05 |
| MRPL54 | 1.37594 | 0.01235 | 0.994376 | 5.00E-05 |
| MT1A | 1.51569 | 0.00795 | 0.886455 | 5.00E-05 |
| NDUFB10 | 1.32475 | 0.0057 | 0.846368 | 5.00E-05 |
| NDUFB7 | 1.38621 | 0.0126 | 0.874481 | 5.00E-05 |
| NOSIP | 0.946929 | 0.03065 | 0.658089 | 5.00E-05 |
| NOTUM | 1.06902 | 0.0386 | 0.931387 | 5.00E-05 |
| NPR1 | 1.48305 | 0.00695 | 0.605153 | 5.00E-05 |
| NUDT2 | 1.13012 | 0.01165 | 0.587554 | 5.00E-05 |
| ORAI1 | 1.44611 | 0.0009 | 0.820078 | 5.00E-05 |
| PLA2G16 | 2.08351 | 0.0001 | 1.12602 | 5.00E-05 |
| PLA2G2A | 3.99554 | 0.0004 | 1.5244 | 0.0011 |
| PLAC9 | 1.41343 | 0.0092 | 1.67037 | 0.0027 |
| PMVK | 1.58439 | 0.00045 | 0.984667 | 5.00E-05 |
| PTH1R | 1.28775 | 0.04995 | 0.588332 | 5.00E-05 |
| PTMS | 1.30996 | 0.02515 | 0.657144 | 5.00E-05 |
| REEP6 | 2.01879 | 0.00025 | 0.888538 | 5.00E-05 |
| RGS3 | 0.913203 | 0.0326 | 0.813494 | 5.00E-05 |
| SLC17A3 | 1.28439 | 0.00345 | 1.70802 | 5.00E-05 |
| SLC22A18 | 1.48428 | 0.0071 | 0.892282 | 5.00E-05 |
| SLC25A47 | 1.16686 | 0.02395 | 1.22306 | 5.00E-05 |
| SLC27A5 | 2.47459 | 0.0004 | 1.30813 | 5.00E-05 |
| SLC6A13 | 2.51526 | 5.00E-05 | 0.769135 | 5.00E-05 |
| SOD3 | 1.33337 | 0.00635 | 0.729647 | 5.00E-05 |
| SPTBN2 | 2.80965 | 5.00E-05 | 0.680224 | 5.00E-05 |
| SRD5A1 | 1.51039 | 0.00015 | 0.833713 | 5.00E-05 |
| TCEA3 | 1.62659 | 5.00E-05 | 0.878422 | 5.00E-05 |
| TFR2 | 1.06447 | 0.02575 | 0.629907 | 5.00E-05 |
| TLE2 | 0.792564 | 0.04675 | 1.03475 | 5.00E-05 |
| TMEM161A | 0.971162 | 0.0397 | 0.619253 | 5.00E-05 |
| TPST2 | 0.889385 | 0.02705 | 0.597177 | 5.00E-05 |
| TUBA4A | 2.00929 | 0.0239 | 0.636691 | 5.00E-05 |
| UPB1 | 1.39654 | 0.0328 | 0.726909 | 5.00E-05 |
