# Supplementary material for: Forkhead box A3 attenuated the progression of fibrosis in a rat model of biliary atresia
Source: Cell Death Dis. 2017 Mar 30;8(3):e2719–. doi: 10.1038/cddis.2017.99 (PMC5386589; doi:10.1038/cddis.2017.99)
Supplement: Supplementary Table 7 [file cddis201799x10.docx]

**Supplementary Table 7**. Primers sequences for quantitative RT-PCR

| Primer | Primer sequence | Size (bp) |
| --- | --- | --- |
| Foxa3 (Homo sapiens)  ([NM_004497](http://www.ncbi.nlm.nih.gov/entrez/query.fcgi?cmd=Search&db=Nucleotide&term=NM_004497)) | F: 5’- GAGATGCCGAAGGGGTATCG -3’  R: 5’- TGATTCTCCCGGTAGTAAGGG-3’ | 164 |
| CTGF (Homo sapiens)  (NM_001901.2) | F: 5’- TAAGGTGTGGCTTTAGGAG -3’  R: 5’- TCTTGATGGCTGGAGAATG -3’ | 183 |
| DAPK1 (Homo sapiens)  (NM_001288729.1) | F: 5’- CAAGACAGGCACGGCAATAC-3’  R: 5’- GGCTCCCATCAGACAGAGATAC-3’ | 186 |
| EGFL7 (Homo sapiens)  ([NM_201446](http://www.ncbi.nlm.nih.gov/entrez/query.fcgi?cmd=Search&db=Nucleotide&term=NM_201446)) | F: 5’- TGAATGCAGTGCTAGGAGGG -3’  R: 5’- GCACACAGAGTGTACCGTCT -3’ | 116 |
| GAPDH (Homo sapiens)  (NM_001256799.1) | F: 5’- CACCCACTCCTCCACCTTTG -3’  R: 5’- CCACCACCCTGTTGCTGTAG -3’ | 110 |
| α-SMA (Rattus norvegicus)  (NM_031004.2) | F: 5’- AACACGGCATCATCACCAAC -3’  R: 5’- CACAGCCTGAATAGCCACATAC -3’ | 203 |
| CTGF (Rattus norvegicus)  (NM_022266.2) | F: 5’-CGTAGACGGTAAAGCAATGG-3’  R: 5’-AGCAGCAAACACTTCCTC-3’ | 140 |
| Collagen I (Rattus norvegicus) (NM_053304.1) | F: 5’- TCAAGATGTGCCACTCTG -3’  R: 5’- ACCTTCGCTTCCATACTC -3’ | 242 |
| Collagen III (Rattus norvegicus)  (NM_032085.1) | F: 5’- GTCCACAGCCTTCTACAC -3’  R: 5’- TCCGACTCCAGACTTGAC -3’ | 233 |
| TGF-β1 (Rattus norvegicus)  (NM_021578.2) | F: 5’-AAGGACCTGGGTTGGAAGTG-3’  R: 5’-TGGTTGTAGAGGGCAAGGAC-3’ | 125 |
| GAPDH (Rattus norvegicus)  (NM_017008.3) | F: 5’- GTCGGTGTGAACGGATTTG -3’  R: 5’- TCCCATTCTCAGCCTTGAC -3’ | 181 |
